# Remarks on the Treatment of the Typhoid State of Fever

**Published:** 1816-05

**Authors:** David Hosack

**Affiliations:** Professor of the Theory and Practice of Physic and Clinical Medicine in the University of the State of New-York.


*For the London Medical and Physical Journal.*

*Remarks on the Treatment of the Typhoid State of Fever;* by DAVID HOSACK, M.D. Professor of the Theory and Practice of Physic and Clinical Medicine in the University of the State of New-York.

FROM the time of Hippocrates to the present day, the subject of fever, more than any other disease to which the human frame is liable, has received the attention of physicians. Yet, looking into our obituaries, we find that fever and febrile diseases still constitute the great outlets to human life, and are at this day almost as fatal as they were in the time of Sydenham, who calculated that fevers, properly so called, make up nearly two-thirds of the diseases which prove fatal to mankind; and that eight out of nine of all who die are cut off by febrile complaints. However minutely, therefore, we may be acquainted with the symptoms of fever in its various forms and stages; however extensive may be our knowledge of its predisposing and exciting causes, we certainly are very deficient in our acquaintance with the *proximate* cause of fever, or its treatment would be more distinctly defined in its various stages, than it appears to be in any of the great practical works that have fallen under our notice. Whence, then, has arisen the variant, and, we may almost say, the *empirical* practice, that fills the pages of the best writers on fevers, and that are even to be found in the truly valuable works of Boerhaave, Cullen, Wilson, Fordyce, and others? We answer—it is, in a great degree, ascribable to the local views to which some of those writers have been limited by their own hypotheses, and by which others have been subsequently enslaved.

Boerhaave's exclusive attention to the *humoral pathology* gave him necessarily but a limited and partial view of the nature of fever, and its operations upon the various parts of the animal economy; he, consequently, neglected all those indications in the treatment, that a more extensive view of the *nervous system*, as taken by Hoffman and Cullen, would have pointed out. But his successor, Cullen, on the other hand, by avoiding Scylla, ran into Charybdis. The nervous system, according to his view, had been too much neglected; but, in restoring it to its merited notice, he again, in a great degree, lost sight of all the other parts of the human frame,* pronouncing the *humoral pathology* in particular a creation of the imagination, and in its application to practice altogether hypothetical.

---

* See preface to his First Lines.

The still more recent writings of Brown, Beddoes, Darwin, Girtanner, Clutterbuck, Rush, and others, have been too successful in spreading these partial views of the human structure, and, consequently, limited pathology of the diseases to which it is liable. Even the learned and elaborate work of Wilson is calculated to diffuse the same erroneous doctrines; nor is the more independent and philosophical Fordyce altogether exempt from this charge, although he professes to be totally guided by facts, regardless of hypotheses. Fever, in the opinion of the writer of these remarks, is a disease of the whole system; it appears no less in all the faculties of the mind than in all the functions of the body; it shows itself in every organ of our frame, and affects every nerve and fibre of our system; the absorbing, the circulating, and excreting systems of vessels, are all affected by it; it shows itself in all the various fluids of the body as well as in the solids: in a word, it is omnipresent; it has no one pathognomonic symptom, but is constituted by a concourse of symptoms, and these variously combined in the various forms that fever assumes, depending upon the causes from whence it proceeds, and the condition of body in which it occurs. If this view of the subject be correct, it will necessarily lead the physician to more extensive principles of practice; it will lead him, at the bed-side of the patient, to pay due regard to the nervous system, and the phenomena it exhibits, and the indications thence arising; but at the same time it will lead him to notice the changes which may be induced in the secretions and excretions, and the circulating mass from whence they proceed. We offer these remarks for the purpose of calling the attention of the reader to the too long neglected pathology of the fluids;* at the same time that we invite the attention of the practitioner to some points of practice, not, in our opinion, sufficiently attended to in the treatment of fevers, and which the successful treatment of some recent cases of typhus fever have enabled us still further to confirm. It is proper here to remark, that, when speaking of fevers, we have in view the continued type of fevers properly so called, not referring to the phlegmasiæ or other pyrexious diseases; yet, in many instances, the principles we wish to inculcate, and the practical deductions thence arising, will be no less applicable in the *typhoid* state of many of the phlegmasiæ, and other fe-

---

* See Dyckman on the Pathology of the Fluids, and the review of the same Dissertation in the Amer. Med. and Phil. Reg. vol. 4.

brile brile diseases, than they are to the advanced stage of typhus fever itself.

It will be acknowledged that fever cannot long continue without inducing debility in the heart and arteries, in common with all the other parts of the system, and that the sensibility to impressions must be proportionally increased. They are, consequently, predisposed to be more readily acted upon even by the natural stimuli of the system; the heart and vessels are accordingly excited to preternatural frequency, even operated upon by the blood and other fluids of the system in their natural and healthy condition, as we see daily illustrated in the progress of all fevers, and in convalescence from fever: We contend that fever, long continued, not only wastes the power of the solids, rendering them more irritable, but by the derangement in the functions and excretions, perhaps by the action of the blood-vessels themselves upon their contents, and especially by the retention of those materials which should have been thrown out of the system as noxious, which in health are constantly ejected, the circulating fluids become changed and vitiated, and thereby become additional sources of irritation to the heart and arteries, whose susceptibility of impression, as we have just observed, is also morbidly increased. From this view of the more irritable state of the circulating system, and the vitiated condition of the fluids, we infer, that unless by some salutary power inherent in the system itself, or by some means suggested by art, the greater irritability of the whole system, and of the heart and arteries in particular, be diminished, or the morbid changes induced in the fluids they circulate be counteracted, these causes of fever, mutually operating upon each other, must increase, and fever be continued until the vital principle itself be totally expended. How far, then, we ask, is the attention of physicians directed to these two cardinal objects, in the treatment of the advanced stage of fevers? how far is their practice calculated either to impart vigour to the system, and thereby to lessen the morbid sensibility of the nervous and moving fibre, or to counteract the septic tendency of the circulating fluids, which obtains in most fevers of the continued type?

Are we not hereby led to condemn that indiscriminate and long-continued use of the *debilitating evacuants*, usually prescribed at this advanced period of fevers and febrile diseases, in as far as they are calculated to add to that waste of excitement, and that very vitiation, to which we have referred? Is not the abstinence, too, usually enjoined by physicians in the typhoid stage of fever, for the same reasons, no less to be reprobated? Are we not led, upon the same

 principle, principle, to condemn the prescription of *camphor*, *musk*, *opium*, *digitalis*, and other powerful sedatives, so frequently directed in this stage of fever? We refer to the ordinary mode and quantity in which these narcotics are administered in fevers by the greater part of practitioners; and who, forsooth, by a strange misnomer, denominate them *stimulants!**

The indiscriminate practice of *purging*, as advised in typhus fevers by Dr. Hamilton,† of Edinburgh, is, in our opinion, no less dangerous by the debility it induces, and is not prescribed with sufficient caution by that distinguished practitioner, for whose opinions and practice, on most occasions, we entertain, and beg leave to express, our highest respect. Even the long-continued exhibition of the various preparations of mercury and antimony, is, in the opinion of the writer, a no less dangerous and fatal practice in this advanced stage of fever. On the contrary, if the views we have taken be correct, after the indications which arise in the first stage of continued fevers have been fulfilled, in the means of accomplishing which most physicians are agreed; after the necessary evacuations by the lancet and other depleting means have been made, which are frequently called for, both in the invasion and in the progress of fever; after the stomach and bowels have been cleansed, and due attention has been given to the no less important function performed by the skin, our attention should next be given to the two following objects, and which the practitioner should never lose sight of when the typhoid state of fever has actually arrived: 1st. To preserve the natural powers of the system, and carefully to guard against every further waste of excitement; 2d. By suitable antiseptic nourishment, and other means, including external as well as internal applications, to preserve the circulating fluids from those morbid changes to which they constantly and rapidly tend in all fevers of the continued type, especially in those arising from *contagion*, which, in a peculiar manner, depresses and exhausts the vital powers. In this advanced or typhoid state of fever, characterized by a disturbed state of the brain and nervous system, showing itself in delirium, watchfulness, or irregular and interrupted sleep, frequent sighing and subsultus tendinum; attended with an increased but feeble circulation, hurried and irregular respiration, with its usual

* For the evidence of the sedative effects of opium, see Dr. Bard's Inaugural Dissertation, Edinburgh, 1765; also Dr. Monroe's Experiments on Opium.

† See his valuable work on the Use of Purgatives.

consequences, consequences, an increased heat of the body and dryness of the surface; characterized, also, by a deranged state of the secretions and excretions, exhibiting themselves in an offensive breath, turbid urine, frothy and offensive discharges from the bowels, a foul sordes about the teeth and gums, discoloured lips, and a brown or a black state of the tongue; and, perhaps, added to these, a cadaverous and offensive smell of the whole body: in this condition of the system, the means of fulfilling the indications before mentioned are, 1st, to supply the patient with the most powerful stimuli, both diffusible and permanent, viz. the volatile alkali, æther, wine,* wine-whey, porter, yeast, bark,† Virginia snake-root, bitters, and the mineral acids, preferring each or either of these according to the peculiar circumstances of the case. We are aware that this practice is reprobated by many physicians as improper in this state of excitement, whatever may be the stage of the disease, or the circumstances that may have induced it. This leads us to observe that many physicians are not sufficiently attentive to discriminate between the *simple excitement* of the early stages of fever, which is characterized by the symptoms of inflammatory action, and is kept up by considerable vigour of the system; and the *complicated excitement*, which appears when the powers of life are greatly exhausted, and the disease has been long protracted. A corresponding want of discrimination appears in their practice; they, therefore, condemn, in the *last* stage, those means of excitement which are injurious in the *first;* and they approve, in the last, the continuance of the same depleting and debilitating means that have been found useful in the first: what! say they, administer *wine*, *bitters*, or *bark* in this quickened circulation, attended with a hot and dry skin? We answer, that, in such typhoid state of body, in this exhausted state of the vital powers, the remedies that have been enumerated are among the most effectual means of reducing that very heat of skin, and of diminishing that increased excitement of the whole system, which, as we have before remarked, are frequently both ascribable to the morbid sensibility of the heart and vessels to their vitiated contents; and that this sensibility being counteracted, the circulation is necessarily reduced in frequency, the respiration becomes less hurried, and that the heat of the system, which

* The reader will find some pertinent practical remarks on the quantity of wine which may be safely and advantageously administered in this stage and character of fever, in Moore's Med. Sketches, p. 13, 517, &c.

† See Moore's Med. Sketches, p. 509.

is is ever in proportion to the circulation and rapidity of respiration is, consequently, diminished.

But, 2dly, We should be no less attentive to the state of the fluids than we are to counteract the morbid excitement of the solids: with this view, attention should be daily given to the bowels for the purpose of evacuating their offensive contents, especially of the lower tract of the intestinal canal; for, these malcontents being retained, not only in some instances become the source of irritation to the intestines themselves, producing diarrhœa, but, by their reabsorption into the mass of circulating fluids, which are thereby rendered still more malignant, they necessarily constitute fresh sources of febrile excitement. Evacuations from the bowels, however, are not to be obtained at that expense of the powers of the whole system, which the means recommended by Dr. Hamilton are calculated to produce; on the contrary, at this advanced period of fever, we should just as readily think of putting a lancet into the patient's arm, as emptying his bowels by the active purges he has directed: these, too, we suppose to have been already administered in the first stages of the disease. Enemata, or, at most, the occasional use of small doses of rhubarb and magnesia, or some other mild cathartic, are only, in our opinion, admissible at this period of the disease. For the united purposes of preserving the surface in a perspirable state, of diminishing its temperature when excessive, and of removing the offensive materials which are excreted by the skin, and constantly accumulated upon it, the body should be regularly cleansed once or twice in the day, by ablutions of vinegar and water, which should be applied, either tepid or cold, according to the temperature of the body;* and should the skin remain dry, after such ablutions have been made, fomentations of vinegar and water applied to the extremities, and steadily persisted in, are among the most effectual means of relaxing the surface, at the same time that they are calculated to allay much of that distressing restlessness which attends this stage of the disease. Upon the same principle of correcting the state of the fluids, the nourishments directed should be exclusively of the *vegetable* kind, as best calculated to resist that putrescent tendency which manifests itself in this state of body: for this purpose, *arrow-root*, *sago*, *tapioca*, *Indian* or *oatmeal gruel*, rendered palatable by the plentiful addition of wine, and some of the most grateful aromatics, should be hourly administered in this exhausted state of the system. The bedding

* See Currie and Jackson on Cold Bathing in Fevers.

and and the dress of the patient, especially if he wear flannel next the skin, which is the preferable clothing in this form of fever, should also be frequently renewed. For the purpose of controlling that restlessness which usually appears in the evening exacerbation, and of procuring sleep, *an occasional anodyne* may, in many instances, be administered with the most beneficial effects; but the indiscriminate use of opium or laudanum, throughout the day, and through the whole progress of the fever, with the view to their supposed stimulant effects, cannot be too severely reprobated; nor have we ever witnessed the stimulant effects ascribed to the fashionable *camphorated julep*, and other preparations of that medicine so often had recourse to; but we can indeed say, that we have, in very many instances, witnessed its debilitating, and, as we believe, its fatal effects, in the typhoid state of fever. Such is the practice the author of these remarks has pursued for many years past in the typhus fever of this city, the typhoid stage of scarlatina, peripneumonia typhoides, and in other febrile diseases; and he can bear the most unequivocal testimony in favour of its safety and success.

